# Intranasal Oxytocin Modulates Decision-Making Depending on Outcome Predictability—A Randomized Within-Subject Controlled Trial in Healthy Males

**DOI:** 10.3390/biomedicines10123230

**Published:** 2022-12-12

**Authors:** Paul Theo Zebhauser, Ana Macchia, Edward Gold, Stephanie Salcedo, Bethany Burum, Miguel Alonso-Alonso, Daniel T. Gilbert, Alvaro Pascual-Leone, Anna-Katharine Brem

**Affiliations:** 1 Department of Neurology, School of Medicine, Technical University of Munich, 81675 Munich, Germany; 2 Clinic for Psychiatry/Psychotherapy III, Ulm University, 89075 Ulm, Germany; 3 Berenson-Allen Center for Noninvasive Brain Stimulation, Beth Israel Deaconess Medical Center, Department of Neurology, Harvard Medical School, Boston, MA 02115, USA; 4 Department of Psychology, Harvard University, Cambridge, MA 02138, USA; 5 Department of Neurology, Harvard Medical School, Boston, MA 02115, USA; 6 Hinda and Arthur Marcus Institute for Aging Research and Deanna and Sidney Wolk Center for Memory Health, Hebrew Senior Life, Boston, MA 02131, USA; 7 Department of Old Age Psychiatry, Institute of Psychiatry, Psychology and Neuroscience, King’s College London, London SE5 8AF, UK; 8 University Hospital of Old Age Psychiatry, University of Bern, 3008 Bern, Switzerland

**Keywords:** oxytocin, intranasal, risk-taking, decision-making, Iowa Gambling Task, Cambridge Risk Task

## Abstract

Oxytocin (OT) has been extensively studied with regard to its socio-cognitive and -behavioral effects. Its potential as a therapeutic agent is being discussed for a range of neuropsychiatric conditions. However, there is limited evidence of its effects on non-social cognition in general and decision-making in particular, despite the importance of these functions in neuropsychiatry. Using a crossover/within-subject, blinded, randomized design, we investigated for the first time if intranasal OT (24 IU) affects decision-making differently depending on outcome predictability/ambiguity in healthy males. The Iowa Gambling Task (IGT) and the Cambridge Risk Task (CRT) were used to assess decision-making under low outcome predictability/high ambiguity and under high outcome probability/low ambiguity, respectively. After administration of OT, subjects performed worse and exhibited riskier performance in the IGT (low outcome predictability/high ambiguity), whereas they made borderline-significant less risky decisions in the CRT (high outcome probability/low ambiguity) as compared to the control condition. Decision-making in healthy males may therefore be influenced by OT and adjusted as a function of contextual information, with implications for clinical trials investigating OT in neuropsychiatric conditions.

## 1. Introduction

Over the last decades, the neuropeptide oxytocin (OT) has attracted substantial attention due to its assumed role as a modulator of a variety of social [1] and behavioral functions [2]. In recent years, researchers have increasingly debated different pathways of intranasal OT into the central nervous system and whether these lead to functionally relevant increases in central OT levels [3,4]. However, despite many unknowns, the administration of intranasal OT is being intensively researched in a range of neuropsychiatric conditions (for an overview see [5]) due to its potential as a therapeutic agent.

Within this context, OT has traditionally been viewed as a “social” hormone, exerting effects on social cognition and social interaction in healthy participants [6,7,8] and patient populations [9,10]. However, recent work views OT as an allostatic hormone (maintaining stability in changing environments by facilitating the anticipation of future needs and flexible behavioral adaptations) that further modulates non-social cognition and behavior [11]. Recently, it has been discussed that OT plays an important role in cognitive processing and functioning [12,13,14], with an emphasis on executive functioning, working memory and, recently, decision-making. This does not come as a surprise, as the prefrontal cortex (PFC)—a central hub in cognitive processing and a structure crucial to executive functioning and working memory [15]—is innervated by OT-synthesizing neurons [16] and densely expresses OT receptors [17,18]. Despite the parallel involvement of the PFC as the neural substrate of key cognitive functions and an OT target site, the influence of OT administration on cognition in general and executive functioning in particular has been surprisingly under-researched. As indicated above, OT is currently being investigated as a pharmacological agent in the treatment of neuropsychiatric disorders, including autism, schizophrenia, post-traumatic stress-disorder and even neurodegeneration and dementia [4]. Impairments in cognitive processing—mainly in the domain of executive functioning—pose a major challenge in neuropsychiatric populations, as they are central to psychopathology, activities of daily living as well as social and workplace behavior [19,20]. In this context, impaired decision-making—for which the ventromedial PFC plays a crucial role as the hub of widespread cortical and subcortical networks [21]—is a common and prominent symptom in various neuropsychiatric conditions [22].

Hence, trying to modulate decision-making by means of OT-administration might offer a way to positively influence cognition and behavior in various neuropsychiatric conditions. However, to our knowledge, only few studies have examined the effects of OT on decision-making and risk-taking. Risk-taking is a central component of the decision-making process and is highly influenced by cognitive and emotional factors. In this context, decision-making with high outcome predictability (low-risk situation) refers to low-ambiguity situations and vice versa. Decision-making processes in these situations are subserved by overlapping and distinct neural substrates evolving around the PFC [23]. One study in healthy participants reported reduced risk-taking after intranasal OT administration in a task assessing risk under known outcome probabilities (high outcome predictability/low ambiguity) [24]. Another recent study in patients with eating disorders and healthy participants found that after intranasal administration of OT, patients with an eating disorder—compared to healthy participants—displayed safer behavior in the Balloon Analogue Risk Task, a computerized risk-taking task that does not provide risk probabilities (low outcome predictability/high ambiguity) [25].

Against this background, we intended to examine and disentangle the effects of intranasally administered OT on non-social decision-making conditions and related cognitive functions. To that end, we focused on a sample in healthy males to keep hormonal variances low. As decision-making might depend on the situational context and outcome predictability/ambiguity [26], we were specifically interested if decision-making is differently influenced by OT administration, depending on these risk-related factors. For this purpose, we used two well-known tasks that have been associated with the ventromedial PFC [27,28], the Iowa Gambling Task (IGT [29]) and the Cambridge Risk Task (CRT [30]), to assess non-social decision-making under low outcome predictability/high ambiguity [31,32] and high outcome predictability/low ambiguity [33], respectively. Thus, our central hypothesis was that OT administration has an impact on decision-making and neuropsychological measures associated with the ventromedial PFC. To further shed light on the effects of OT on related cognitive abilities, we employed additional neuropsychological tasks to assess the information-processing bias for positive and negative stimuli, rule acquisition and set-shifting ability.

## 2. Materials and Methods

### 2.1. Study Design

The present study was part of a larger project examining the effects of intranasally administered OT on different behavioral metrics in healthy males [34]. Due to the overall design of the project, participants of the present study had three randomized study visits (separated by at least 7 days), of which one was the experimental condition (active OT, see below for protocol of OT administration) and two were summarized as the control condition. Thus, repeated administration of all behavioral tasks was separated by at least 7 days for all participants, minimizing possible test–retest effects. This was especially important for the IGT, for which data on test-retest reliability is inconclusive [35,36,37]. All visits were counterbalanced, and participants were blinded with respect to whether they received the placebo or OT.

In the experimental condition (OT active, participants blinded to condition), all participants received OT and then waited for 45 min before completing the study tasks, when 24 UI of intranasally administered OT was expected to be most effective [38]. During the waiting period, subjects watched a nature movie and had no human interaction. On the other two visits (control condition; placebo or inactive OT), participants were randomly assigned to receive intranasal OT or placebo in a double-blinded within-subject cross-over design. All tasks reported in this study were then administered after 75 min (after the CNS effects of OT were likely to be diminished or even absent; [38]). To control for the potential remaining effects of OT 75 min after administration, we compared the two control conditions (study tasks administered either 75 min after placebo administration or OT administration; see below). Here, we did not observe differences between the two control conditions (see results section), thus confirming the validity of our study design. During these two visits, which served as the control condition, further unrelated behavioral tasks [34] were administered.

### 2.2. Participants

In the present study, a total of 24 healthy males (mean age: 22.58 ± 3.5 years) participated. The exclusion criteria for participation were any current or past history of psychiatric illness, any unstable medical condition, smoking, nasal pathologies, and abuse of drugs or alcohol. Subjects were instructed to abstain from food for 9 h, from drink (other than water) for two hours, and from alcohol and caffeine for 24 h before study visits. Study visits started at 9 am. Before each study visit, participants were reminded of described intake limitations via phone calls. The study was approved by the local Institutional Review Board (Committee on Clinical Investigations, CCI, 000265) and cede-reviewed by the Institutional Review Board of Harvard University. All participants gave written informed consent prior to study onset according to the Declaration of Helsinki.

### 2.3. Substance Administration

Participants received a single dose of 24 IU OT (Syntocinon Spray, Novartis, Basel, Switzerland); 3 puffs per nostril (each puff containing 4 IU of OT) were administered by a research nurse following a strict protocol. Participants were instructed to close one nostril while the spray was delivered to the other nostril at a 45 degree angle; administration was alternated between the nostrils (15–20 s between each puff). Upon delivery of the spray, participants were instructed to breathe in lightly and avoid inhaling too strongly (to avoid swallowing). To avoid olfactory effects other than those experienced from OT, the placebo contained all the inactive ingredients except the neuropeptide. OT was administered by a nurse, who further assessed vital sign measurements (oral temperature, blood pressure and pulse rate) before and after OT administration.

### 2.4. Decision-Making/Risk-Taking Tasks

The IGT and the CRT were used to assess decision-making under low outcome predictability/high ambiguity and under high outcome probability/low ambiguity, respectively. All tasks were completed at the end of the control visit (control condition) as well as 45 min after administration of OT on the active visit (active condition with OT).

*Iowa Gambling Task (IGT)*: The IGT [29] is a well-studied task that assesses reward-related decision-making under conditions of uncertainty. In this task, participants are asked to pick cards from four virtual decks presented on a computer screen and, as a result, can win or lose game money. Two decks (‘bad card decks’) provide relatively high immediate rewards with the turning of each card, but even higher losses with the turning of some cards, resulting in an overall net loss for every 10 cards turned. The other two decks (‘good card decks’) combine moderate rewards with the turning of each card and relatively small losses with the turning of some cards, resulting in an overall net gain for every 10 cards turned. To optimize gains, it is necessary to figure out, over time, which decks are ‘good’ and which are ‘bad’. Our outcome measure was the ’net score’ (difference score between the overall proportion of good/advantageous decks and bad/disadvantageous decks). Furthermore, we compared the number of chosen decks between conditions.

*Cambridge Risk Task (CRT)*: The CRT [30] is a paradigm that measures reward-related decision-making under known outcome probabilities. Subjects are presented with six horizontally arranged boxes that are colored pink or blue. The ratio of pink and blue boxes varies from trial to trial (5:1, 4:2 or 3:3). Participants try to earn as many points as possible by betting on the color of the box that hides the winning token. The token is equally likely to be hidden in any of the boxes. Therefore, for each trial, the ratio of pink to blue boxes determines the probability of finding the winning token and thus the level of risk. Participants either gain or lose points depending on whether they choose a correct or incorrect color. The amount of points associated with the two colors is indicated on the screen and varies for each trial (90:10, 80:20, 70:30 or 60:40; referred to as balance of reward). Importantly, the largest reward goes together with the least likely—and thus, riskier—of the two options (e.g., a level of risk of 5 (pink): 1 (blue) box is coupled with a balance of reward of 10 (pink): 90 (blue) points). Our outcome measure was the number of choices of the more probable, low-risk option. Additionally, we examined the mean choice reaction time (deliberation time) of the CRT.

### 2.5. Neuropsychological Tasks

*Affective Go/No-go task (AGN)*: The AGN is a task from the CANTAB test battery (http://www.cambridgecognition.com, accessed on 1 June 2022) and assesses information-processing biases for positive and negative stimuli. The test consists of several blocks, each of which presents a series of words from two different affective categories: Positive (for example, joyful) and Negative (for example, hopeless). The participants are given a target category (positive or negative) and are asked to press the press pad when they see a word matching this category.

*Intra-Extra Dimensional Set Shift (IED)*: The IED is a task from the CANTAB test battery and assesses rule acquisition and reversal. Two artificial dimensions are used in this test: color-filled shapes and white lines. Simple stimuli are made up of just one of these dimensions, whereas compound stimuli are made up of both, namely white lines overlying color-filled shapes. The participant starts by seeing two simple color-filled shapes and must learn which one is correct by touching it. Feedback teaches the participant which stimulus is correct, and after six correct responses, the stimuli and/or rules are changed. These shifts are initially intra-dimensional (e.g., color filled shapes remain the only relevant dimension) and, later, extra-dimensional (white lines become the only relevant dimension). Participants progress through the test by satisfying a set criterion of learning at each stage (6 consecutive correct responses). If at any stage the participant fails to reach this criterion after 50 trials, the test terminates. On each visit, subjects were furthermore asked whether they believed to have received the drug or the placebo. Side effects were assessed before and after each visit.

### 2.6. Analysis

Data were analyzed using JASP Version 0.15 (Amsterdam, The Netherlands) [39]. Normality was inspected with the Shapiro-Wilk test and visual distributions. Non-normal data (low risk choices CRT, difference score of the IGT/CRT) were additionally investigated using non-parametric tests and were only reported if we found deviations in statistical significance. Due to drop-out (n = 3) and technical problems (CRT n = 1), the sample size decreased to 21 individuals in the IGT and 20 individuals in the CRT. For all tests, significance was set using a 95% confidence interval (α = 0.05). Group analyses and post hoc comparisons were calculated with repeated-measure ANOVAs (Type III Sum of Squares) and Student’s *t*-tests, respectively. Pairwise post hoc ANOVA comparisons were adjusted with Holm correction for multiple testing and the assumption of sphericity was assessed by Mauchly’s test. Correlations were calculated using Pearson’s *r* tests. Beliefs about drug administration were assessed with Pearson’s chi-square test. We evaluated potential differences in outcome measures between control conditions (study tasks administered either 75 min after placebo administration or OT administration) by calculating *t*-tests. We complemented frequentist analysis with Bayesian data analysis using the equivalent statistical procedures (Bayesian paired samples *t*-test, Bayesian repeated measures ANOVA, Bayesian correlation) for a more detailed understanding of the evidence for the alternative hypothesis. The Bayes factor (BF_10_) was used to quantify effects in this context and was interpreted as ‘strong’, ‘moderate’, or ‘anecdotal’ evidence according to recent guidelines [40].

## 3. Results

In Table 1, raw data for the decision-making tasks (IGT and CRT) in the experimental and control condition are presented.

### 3.1. Decision-Making

**Figure 1:**
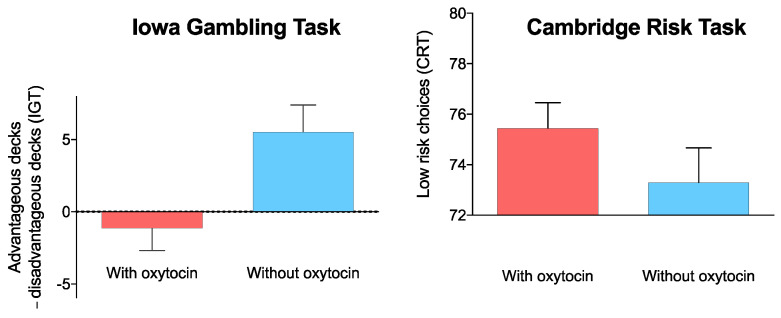
Dissociation between the IGT and the CRT. After intranasal administration of OT, participants chose significantly more risky decks in the IGT, as opposed to decreased risk-taking in the CRT (indicated by higher scores; values in both panels correspond to mean ± standard error [SE]).

Paired *t*-tests revealed significant differences (‘strong’ evidence) in the IGT net score (*t*(20) = −3.24, *p* = 0.004, *d* = −0.71; 95% CI (−10.99 to −2.39), BF_10_ = 10.76, Mdn = −0.64; 95% CI (−1.12, −0.18)). Furthermore, we found a borderline effect (‘anecdotal’ evidence) for a decrease in low-risk choices of the CRT (*W* = 116, *p* = 0.063, *r_b_* = 0.516, BF_10_ = 1.56, Mdn = 0.43; 95% CI (−0.01, 0.88)). Moreover, the deliberation time in the CRT did not differ between the OT and placebo visit (Table 1). Our data shows that it is 10.76 times more plausible that participants yielded lower IGT net scores in the OT condition as compared to the no-OT condition, whereas there is ‘anecdotal’ evidence for a null effect of the CRT low-risk choices (BF_10_ = 1.56). This finding indicates that intranasal administration of OT had an effect on IGT performance, while it had a borderline effect on choice behavior in the CRT (Figure 1). Furthermore, we conducted paired-sample *t*-tests/Bayesian paired-sample *t*-tests to compare the number of choices for single decks between conditions. We found significantly more choices (‘moderate’ evidence) of deck B in the OT condition, indicating riskier choices (*t*(20) = −2.78, *p* = 0.011, *d* = −0.36; BF_10_ = 4.51). For decks A and D, the number of choices did not differ significantly between conditions using frequentist statistics (*p* > 0.0.5), while a trend of ‘anecdotal’ evidence was found for deck C (*t*(20) = 2.05, *p* = 0.054, *d* = 0.45; BF_10_ = 1.28) showing that this deck (the safest of the four decks) was less frequently chosen. Performance in decision-making tasks did not differ for different control conditions (75 min post placebo vs. 75 min post active OT; *p* = 0.566 for the IGT, *p* = 0.318 for the CRT; see methods section) confirming the reliability of the control conditions.

**Figure 2:**
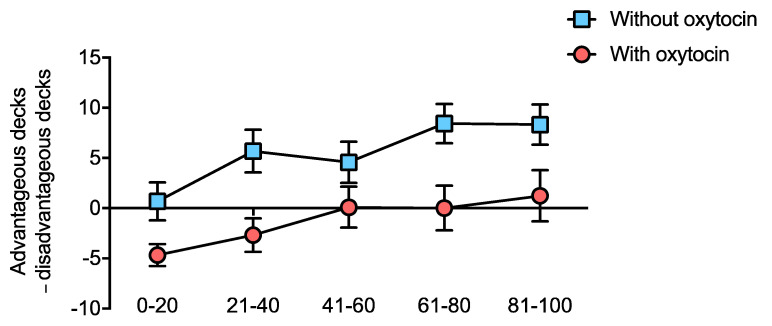
IGT net-scores over decks with and without oxytocin. Results are depicted in groups of 20 decks over time (100 decks in total). Differences between visits (with/without OT) revealed significant main effects for Condition and Time. Participants made significantly more risky decisions with OT, while the learning curve was highly similar between conditions (indicated values correspond to mean ± standard error (SE)).

Further, we were interested in the time course of the effect on the IGT, as has been described in previous studies [29,41,42]. According to this previous research, we divided the results (100 decks) into five blocks with 20 decks each. A repeated measures 2 × 5 ANOVA (within-subject factors: Condition (OT, No-OT), Block (1–5)) did not show a significant interaction (F (4,80) = 1.00, *p* = 0.410, η^2^_p_ = 0.048), which indicates that the time course, which reflects the learning curve, remained the same independent of OT intake (Figure 2). Visual inspection of the average learning curve (net score, Figure 2) in the IGT showed a similar slope and magnitude of effect compared to other studies in healthy samples [43,44,45]. The main effects on Condition (F (1,80) = 10.67, *p* = 0.004, η^2^_p_ = 0.348) and Block (F (4,80) = 8.31, *p* < 0.001, η^2^_p_ = 0.293) were significant. Post hoc comparison revealed that individuals scored higher on the IGT in the third (*p* = 0.022), fourth (*p* < 0.001) and fifth (*p* < 0.001) block, and a borderline higher effect was seen in the second block (*p* = 0.065) as compared to the first block. As expected, the dependent variable increases as a function of block as suggested by a linear trend (F (1,20) = 20.82, *p* < 0.001, η^2^_p_ = 0.510). After OT administration, subjects thus tended to make riskier choices in the IGT and continued to do so throughout the task. OT-associated differences in the IGT and CRT were not correlated (r = 0.079, *p* = 0.739; 95% CI (−0.38 to 0.50)), BF10 = 0.29). Hence, increased risk-taking in one task was not associated with decreased risk-taking in the other.

### 3.2. Emotional Valence (AGN) and Rule Acquisition (IED)

We ran a repeated-measure ANOVA for AGN omissions with Condition (OT, No-OT) and Valence (Positive, Negative) as within-subject factors. The main effects of Condition and Valence were not significant (Condition: F (1,19) = 1.71, *p* = 0.206, η^2^_p_ = 0.083; Valence: F (1,19) = 0.01, *p* = 0.925, η^2^_p_ < 0.001). The interaction Condition × Valence failed to reach significance (F (1,19) = 2.09, *p* = 0.164, η^2^_p_ = 0.099). The repeated measures ANOVA for AGN latencies (time until response) with Condition (OT, No-OT) and Valence (Positive, Negative) as within-subject factors showed no main effect of Condition and Valence (Condition: F (1,19) = 0.16, *p* = 0.693, η^2^_p_ = 0.008; Valence: F (1,19) = 1.58, *p* = 0.225, η^2^_p_ = 0.077) and no interaction of Condition × Valence (F (1,19) = 1.90, *p* = 0.184, η^2^_p_ = 0.091). A paired t-test revealed no differences in IED errors between conditions (OT, No-OT) (*t*(19) = 1.24, *p* = 0.229, *d* = 0.278; 95% CI (−0.75 to 2.95), BF_10_ = 0.46, Mdn = −0.24; 95% CI (−0.67, 0.17)). The very low number of errors in both the AGN and the IED might indicate a ceiling effect.

### 3.3. Side Effects

Side effects were assessed before and after each visit. These included ‘Irritation of the nasal mucosa’, ‘Nausea’, ‘Headache’, ‘Change in mood’, ‘Allergic dermatitis’, and ‘Other’. No serious adverse events occurred during the study. One subject was excluded due to nose bleeding before the visit and replaced by a new participant.

When subjects were asked about their beliefs of whether they received OT or the placebo on the first two double-blinded visits, 65.22% of subjects correctly guessed they received the real drug and 63.64% correctly guessed they received the placebo. There was a trend association between the condition and the belief (χ^2^(1) = 2.68, *p* = 0.076). Correctly guessed OT administration was mostly associated with changed alertness levels (increased as well as decreased) and the smell/taste of the drug. However, subjects who wrongly thought they received OT mentioned the same reasons. We therefore conclude that our blinding procedure was successful.

## 4. Discussion

We found a dissociation between OT-associated effects on the IGT and the CRT, which measure risk-taking under low outcome predictability/high ambiguity and under high outcome probability/low ambiguity, respectively. Participants made significantly more risky decisions (‘strong’ evidence) and more frequently chose ‘riskier’ decks (‘moderate’ evidence) after intake of OT in the IGT, a task using unknown probabilities (high ambiguity) as opposed to borderline-significant (‘anecdotal’ evidence) less risky decisions in the CRT, in which probabilities were known (low ambiguity). In line with our results, a recent study in healthy participants found reduced risk-taking after intranasal OT administration in a task assessing risk under known outcome probabilities [24]. Adding to the literature, we were able to disentangle OT effects depending on situational ambiguity and risk load. Another recent study [25] in patients with eating disorders found that after intranasal administration of OT, patients with eating disorders—compared to healthy participants—displayed less risky behavior in the Balloon Analogue Risk Task, which can be classified as a risk-taking task with unknown but, to a certain degree, learning-dependent probabilities [46]. This result does not concur with our findings of increased risk-taking in situations with high ambiguity. However, study results are comparable only in a very limited manner, as the mentioned study focused on a psychiatric sample. Additionally, the Balloon Analogue Risk Task assesses risk-taking using a different experimental paradigm compared to the IGT [46].

The finding of a dissociation between OT-associated effects on risk-taking depending on situational ambiguity might reflect the importance of OT in evaluating contextual factors in complex situations and the guidance of strategies during abstract, non-social decision-making. Our results further suggest that OT possibly enhances primed behavioral tendencies such as are present in the IGT (high-risk context) and CRT (low-risk context), respectively. In this context, OT might exert an amplifying role regarding risky behavioral tendencies while exerting contrary effects in low-risk situations. Another possible explanation for our results might be that OT decreases inhibitory control in riskier and more emotion-driven contexts—eventually leading to impaired learning, which could be reflected by worse performance in the IGT after OT administration. Related, recent studies have shown modulatory effects of OT on inhibitory control [47,48]. Though not further explored in the present study, we believe that the ventromedial PFC is key to OT-induced modulation of decision-making and might have mediated the observed effects. Our study tasks were chosen as they are known to be associated with the ventromedial PFC [27], which is strongly associated with behavior that is affected by exogenous administration of OT. Moreover, the ventromedial PFC has been directly linked to OT-induced behavioral changes in autism [9]. Furthermore, the ventromedial PFC is specifically involved when decisions are made in an ambiguous or risky context, both during risky decision-making under high ambiguity such as in the IGT [41,49] as well as risky decision-making under low ambiguity and known outcome probabilities, such as in the Cambridge Gamble Task [50].

Regarding our findings of OT affecting performance in the IGT, the possible role of learning should be mentioned. In this context, we cannot rule out that OT might have impaired learning, resulting in lower net scores. However, we consider this unlikely given the similar learning curves (see Figure 2) for both conditions. In addition, we did not find any differences in rule acquisition and reversal (as assessed with the IED) after administration of OT.

The modulation of decision-making by means of intranasal OT has implications for OT research in the field of neuropsychiatry. As outlined above, the administration of OT has been extensively discussed as a promising treatment for various diseases [4], and impaired decision-making is a hallmark of cognitive impairment in a range of conditions [22]. Future studies should investigate OT-driven modulation of decision-making in psychiatric populations like those diagnosed with anxiety and depression [51], schizophrenia [52] and neurodevelopmental disorders like autism spectrum disorder [53] or attention-deficit/hyperactivity disorder [54] as well as the possible effects on symptom expression and facilitation of therapeutic progress.

### 4.1. Limitations

The inclusion of only male participants poses an important limitation of our study. Generalization of results is not possible, especially as OT is a highly sex-dependent hormone. Thus, diverging results of our experimental protocol in a female or mixed sample cannot be excluded.

To our knowledge, this is the first study to explore immediate OT-induced effects on decision-making under varied situational ambiguity. Though we administered widely used tasks for this study, it would be desirable to develop and employ tasks using the same design and only varying situational ambiguity (risk load). In this context, it must be noted that decision-making processes, especially in the IGT, may be influenced by additional factors. It has been suggested that the IGT assesses ‘hot’ or emotional decision-making processes [55]. This is consistent with the ‘somatic marker’ hypothesis, which proposes that emotion-based biasing signals arising from the body, which are processed in the emotion circuitry of the brain evolving around the ventromedial PFC, are crucially involved in decision-making in situations marked by complexity and uncertainty [56]. It should be noted that our protocol of OT administration is debatable, as time- and dosage-effects of intranasally delivered OT are currently heavily debated [4]. While the frame of 45–70 min after administration seems to be a suitable window to investigate behavioral effects of OT [38], it can be argued that lower doses than 24 IU elicit higher neural (right amygdala fMRI activation) and behavioral responses [4,57].

Due to the study design, we cannot rule out that the administration of additional (facial emotion recognition) tasks during the control condition could have influenced behavioral outcomes. However, as we found opposing effects for the two tasks within the same session, we believe that such a potential confounder—if present at all—did not have large effects. Another critical matter of our study design is the composite character of the control condition. Due to the design of the main project, we combined control conditions 75 min after placebo administration and 75 min after administration of OT based on the assumption that OT effects on decision-making at this point would be clearly reduced or absent. We validated this assumption by comparing outcome measures between our different control conditions. Furthermore, the validity and generalizability of our study results are limited by small sample size.

### 4.2. Outlook

These results should be replicated in a larger and more diverse sample. Importantly, similar study designs should be used in female or mixed-sex samples, as our study was conducted in a sample of male participants only. In addition, neural correlates of OT-induced effects on decision-making and risk-taking should be explored by means of fMRI and/or EEG. In recent years, the effects of oxytocin on neuronal networks and connectivity [58,59] have been uncovered. In the context of cognitive functioning and decision-making, mesolimbic [60] and frontal [61] regions (especially, the ventromedial PFC [27,62]) are believed to play an important role, which should be evaluated in respective studies.

Furthermore, the effects of OT on a wider range of cognitive abilities, especially those underlying decision-making and risk-taking (executive functioning, working memory) need to be explored to improve understanding of the exerted mechanisms of OT on cognitive processing and the investigation of the different contributions of emotional and cognitive aspects of decision-making under known risk versus ambiguity. Finally, OT receptor polymorphisms are associated with a large range of behavioral, social and emotional outcomes [63] and could be taken into consideration when devising clinical trials using OT. Finally, a deeper understanding of factors determining OT-induced effects might help researchers to devise individualized treatment approaches for patient populations.

## 5. Conclusions

In summary, the finding of a dissociation between OT-associated effects on risk-taking depending on situational ambiguity might reflect the importance of this neuroactive hormone in the evaluation of context in complex situations and the guidance of strategies during abstract, non-social decision-making. Our results show that OT-induced effects in decision-making are state-dependent and adjusted as a function of current contextual information. This has potential implications for therapeutic applications of intranasal OT in the field of neuropsychiatry, as in various neuropsychiatric conditions, cognitive abilities in general and decision-making are impaired.

## Figures and Tables

**Table 1:** Mean and standard deviations of raw data (decision-making-tasks) for the two conditions.

	OT Condition	Control Condition	*p*-Value	BF_10_
IGT (net score)	−1.16 ± 6.98	5.52 ± 8.51	**0** **.004**	10.76
CRT (low-risk choices) *	75.45 ± 4.50	73.30 ± 6.08	0.063	1.56
CRT (deliberation time (millisec.))	1588.12 ± 520.86	1537.14 ± 536.85	0.705	0.248

IGT: Iowa Gambling Task; CRT: Cambridge Risk Task; * non-parametric test (Wilcoxon signed rank). Bold font indicates *p* < 0.05.

## Data Availability

The data presented in this study are available on request from the corresponding author.
